# Object Transportation by Two Mobile Robots with Hand Carts

**DOI:** 10.1155/2014/684235

**Published:** 2014-10-29

**Authors:** Takuya Sakuyama, Jorge David Figueroa Heredia, Taiki Ogata, Tatsunori Hara, Jun Ota

**Affiliations:** Research into Artifacts, Center for Engineering (RACE), The University of Tokyo, 5-1-5 Kashiwanoha, Kashiwa, Chiba 277-8568, Japan

## Abstract

This paper proposes a methodology by which two small mobile robots can grasp, lift, and transport large objects using hand carts. The specific problems involve generating robot actions and determining the hand cart positions to achieve the stable loading of objects onto the carts. These problems are solved using nonlinear optimization, and we propose an algorithm for generating robot actions. The proposed method was verified through simulations and experiments using actual devices in a real environment. The proposed method could reduce the number of robots required to transport large objects with 50–60%. In addition, we demonstrated the efficacy of this task in real environments where errors occur in robot sensing and movement.

## 1. Introduction

The use of mobile robots for transporting objects in domestic environments is expected to become widely applicable. Mobile robots capable of transporting large objects such as shelves or tables would reduce human labor to a great extent. The size of domestic robots, however, is limited by the narrow spaces of indoor environments. Thus, using small mobile robots to transport large objects is a critical problem in domestic environments. To reduce the weight, humans frequently transport large objects using hand carts, which are effective tools for this purpose.

Several methods exist for enabling multiple mobile robots to cooperate in grasping, lifting, and transporting objects [1–8]. In these methods, the upper transportable weight limit is the sum of the payloads of the mobile robots. However, because that of a single small mobile robot is low, large objects require many mobile robots. Such an implementation is unrealistic, both economically and in terms of control simplicity. Yonezawa et al. [9] developed multiple mobile robots with special mechanisms for automobile transport, but their system cannot be generalized for transporting other objects.

Other researchers have presented methods for pushing, rather than lifting, objects using mobile robots [10–12]. In this way, the robots are not required to support the entire weight of the object, and the acting force during transport is reduced. However, this benefit is negated if significant friction occurs between the object and the floor. Manipulation research has led to a methodology called graspless manipulation [13–15], in which objects are not grasped but are instead manipulated by actions such as rolling, tilting, and sliding, while the object maintains contact with the environment. Again, these methods prevent the robot from bearing the full weight of the object and reduce the force imparted by manipulation. However, this approach has yet to be adopted in mobile robots. Although Yamashita et al. [16] applied graspless manipulation to mobile robot control, their study focused on changing the position of an object, and the transport problem was ignored.

Multiple mobile robots have been used to simultaneously transport or align multiple objects using tools such as rods or ropes [17–19]. However, because the objects remain in contact with the floor, the transport of large objects in the presence of significant friction remains problematic.

As demonstrated in the studies mentioned above, using a team of small mobile robots to lift and transport large objects presents a significant challenge. Moreover, the friction between the object and floor surface becomes important when an object is pushed rather than lifted. In other words, an effective method for large object transport by small mobile robots has yet to be realized.

This paper proposes a method by which two small mobile robots can transport large objects using hand carts. The goals of the study are stated below.

- Restrict the surface area of each robot to less than 1.0 m × 1.0 m.
- Halve the number of robots required for normal transport. Here, the number of robots required for normal transport is defined as the object mass/payload per robot rounded to the nearest integer. Our goal is to transport objects with a mass exceeding four times the payload of a single mobile robot.

This paper extends a previous study [20] by adding the results of (a) simulations of more complicated objects and (b) experiments to verify the applicability of the proposed algorithm to the real world by developing hardware systems for object manipulation and environmental sensing.

This paper is structured as follows.  Section 2 summarizes the proposed method. A method for solving the robot movement is developed in Section 3, and we show how its validity was verified in a simulation.  Section 4 discusses how a robot system was constructed and validated in an experiment. The conclusions are presented in Section 5.

## 2. Overview of Proposed Method

**Figure 1:**
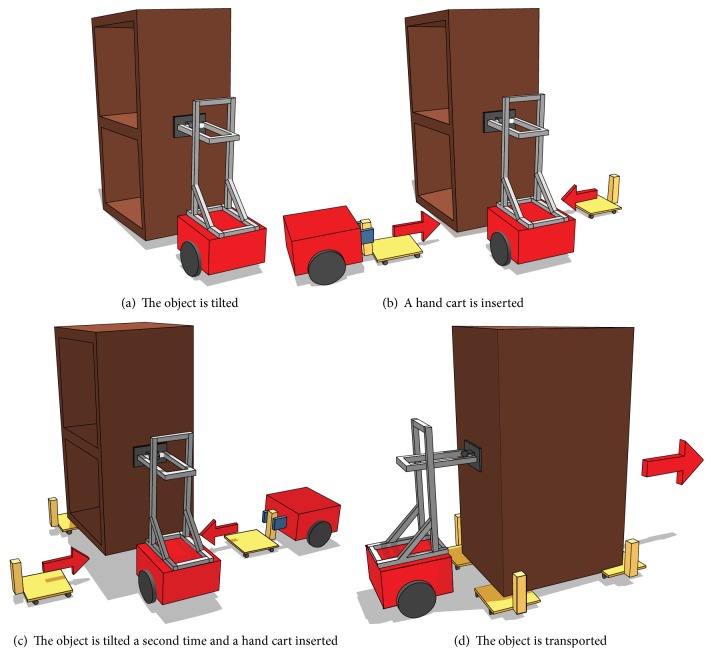
Proposed method of transport by hand carts.

With this method, an object is loaded onto multiple hand carts and transported by two robots: a gripping robot and tilting robot. The gripping robot is equipped with grippers for grasping the object, while the tilting robot possesses an end effector that tilts the object by pushing it. The object is loaded onto the hand cart using the following steps. (1) The tilting robot pushes and tilts the object (Figure 1(a)). (2) The gripping robot inserts the hand cart into the space created below the tilted object (Figure 1(b)). (3) Steps (1) and (2) are repeated until all the hand carts are inserted beneath the object (Figure 1(c)). (4) The tilting robot transports the object by pushing it (Figure 1(d)).

Because the object is transported atop a hand cart, the proposed method requires less force than methods that simply push an object across the floor. Moreover, because the mobile robots can load the object onto the hand cart simply by tilting it, only a small force is required for loading. Thus, the method is suitable for transporting large objects.

The challenges in this method include robot motion planning for object manipulation and the implementation of the robots. The former refers to where the robot should push on the object to tilt it (which depends on the shape of the object), where the hand cart should be inserted beneath the object, and the procedures for implementing these actions. To approach this problem, we formulate an optimization problem that considers robot movement procedures and adopts a multistart local search. For the robot implementation, we must design end effectors that can tilt a large object by pushing it and also determine the correct positions of the robots, object, and hand carts. Here the end effector incorporates a ball screw drive linear motion mechanism. The positioning method adopts a signature of histograms of orientations (SHOT) descriptor, as well as the Hough voting and iterative closed point (ICP) algorithms.

## 3. Planning of Hand Cart Insertions

### 3.1. Problem Statement

The insertion of a hand cart is formulated as described below. First, we assume the following.

- All movements can be assumed to be quasistatic processes.
- The target object is a polyhedron.
- The shape, mass, and center of gravity of the object are known.
- The target object contacts the end effector and hand cart.
- The friction coefficients between the object and floor and between the object and end effector are uniform.

The design variables for this problem are the insertion point of the hand cart (set in advance for *n* carts) and where to push and tilt the object while inserting the hand cart (hereafter termed the end effector pushing point). In other words, this problem constitutes two design problems: determining the hand cart insertion position and identifying the end effector pushing point. Because the robot does not grasp the object tightly in our method, we evaluate the method in terms of its susceptibility to disturbance. To this end, we specify the following objective functions: (a) the object stability in the final state and (b) object stability during hand cart insertion.

### 3.2. Quasistatic Analysis of Tilting Process for Object

First, we conduct a quasistatic analysis of the tilting processes for an object using an end effector for a point contact with friction.

**Figure 2:**
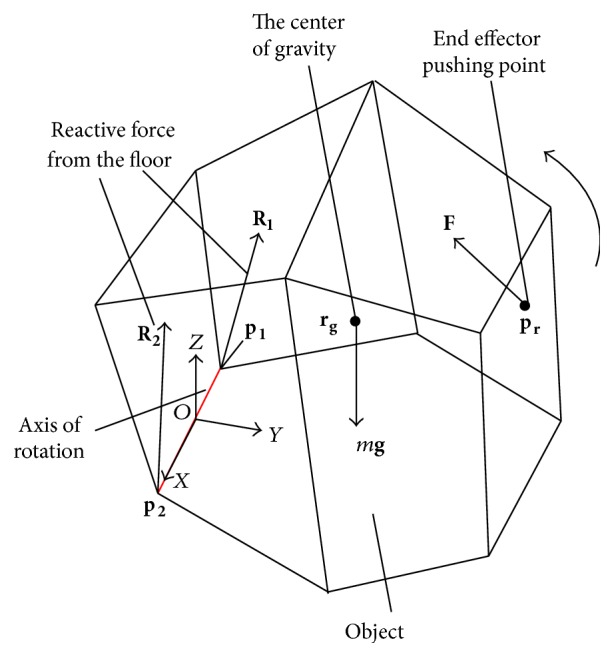
Determining end effector pushing point.

As shown in Figure 2, the origin of the coordinate system is set at the center of the tilted edge. The force on the object and the pushing position are expressed as follows.

(i) **F** = (*f*_*x*_, *f*_*y*_, *f*_*z*_)^*T*^: end effector acting force
(ii) **P**_**r**_ = (*p*_*rx*_, *p*_*ry*_, *p*_*rz*_)^*T*^: end effector pushing position
(iii) **r**_**g**_ = (*r*_*gx*_, *r*_*gy*_, *r*_*gz*_)^*T*^: center of gravity of the object
(iv) *m*: object mass
(v) **g** = (0, 0, −*g*)^*T*^: gravity vector
(vi) **p**_1_ = (*p*_1*x*_, 0, 0)^*T*^, **p**_2_ = (*p*_2*x*_, 0, 0)^*T*^: end points of the axis of rotation
(vii) **R**_1_ = (*R*_1*x*_, *R*_1*y*_, *R*_1*z*_)^*T*^, **R**_2_ = (*R*_2*x*_, *R*_2*y*_, *R*_3*z*_)^*T*^: reactive force exerted on object by the environment.

There is a line of contact between the floor and object during rotation. To simplify the calculation, we replace this continuous contact with the points of contact at the two endpoints of the contact line. The origin of the coordinate system is midway between these two endpoints. The *x*-axis lies in the direction connecting the endpoints, and the *y*-axis lies perpendicular to the *x*-axis in the floor plane.

The following formulae are imposed:

(1) $$\begin{array}{c} F+R_{1}+R_{2}+mg=0, \end{array}$$

(2) $$\begin{array}{c} p_{r}\times F+p_{1}\times R_{1}+p_{2}\times R_{2}+r_{g}\times mg=0. \end{array}$$

Here, the end effector acting force is exerted perpendicularly to the axis of rotation. In other words, if *f*_*x*_ = 0, the *x* component of (1) is

(3) $$\begin{array}{c} R_{1x}+R_{2x}=0. \end{array}$$

In (3), the *x* component of the floor reaction force is purely internal. Here, we regard such an internal force as infeasible in real environments, and we assume the following:

(4) $$\begin{array}{c} R_{1x}=R_{2x}=0. \end{array}$$

The method used to control the end effector is an important issue. For example, in [2], a kind of force control is applied to support the transported object. In this case, however, we assume that the tilting robot end effector is controlled by position control from the viewpoint of simplicity (please note that only one robot exerts a force on the object). In this control, the force is applied in the direction in which the pushing point moves. During tilting, assuming that the object and end effector do not slip (the conditions for the occurrence of slippage will be discussed later), the pushing point of the end effector moves in an arc about the axis of rotation of the tilt. Then, the end effector pushing point necessarily moves in the same direction. Thus, we obtain the following constraining condition:

(5) $$\begin{array}{c} F\cdot P_{ryz}=0. \end{array}$$

Here, **P**_**ryz**_ is an orthogonal projection of **P**_**r**_ on the *yz* plane.

From (1), (2), (4), and (5) and given a parameter **P**_**r**_ = (*p*_*rx*_, *p*_*ry*_, *p*_*rz*_)^*T*^, the forces on the object (**F**, **R**_1_, and **R**_2_) are uniquely determined. In other words, determining the end effector pushing point **P**_**r**_ uniquely specifies the forces acting on the object.

### 3.3. Approach and Details of Planning Algorithms

**Figure 3:**
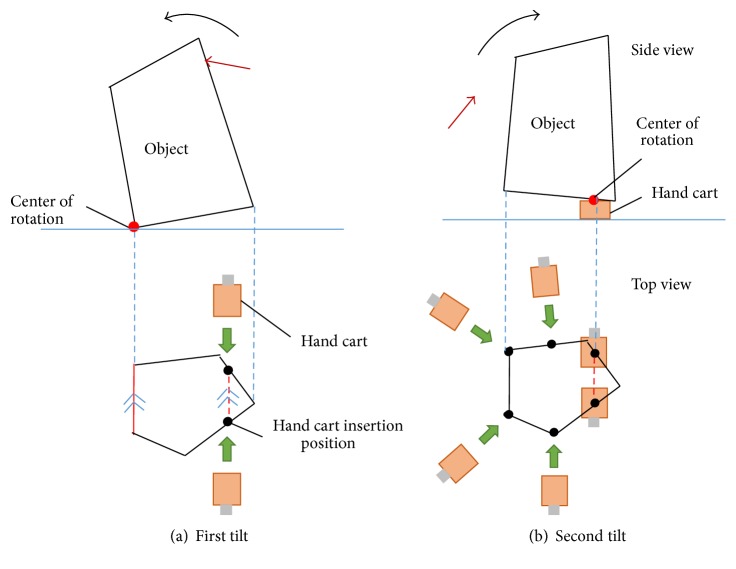
Hand cart insertion procedure.

The problem discussed in Section 3.1 is solved by considering the motion sequence of the robots. First, to minimize the number of actions the robot must perform on the object, and to increase the probability of success, we specify a limit of two object tilts. In addition, during the first tilt, we simultaneously insert two hand carts parallel to the rotation center of the first tilt (Figure 3). Parallel insertion provides stable support to the object once the insertion is complete. The remaining hand carts are inserted during the second tilt.

The number of hand carts in each tilt is obtained using the procedure mentioned above, along with the insertion sequence for the hand carts.

**Figure 4:**
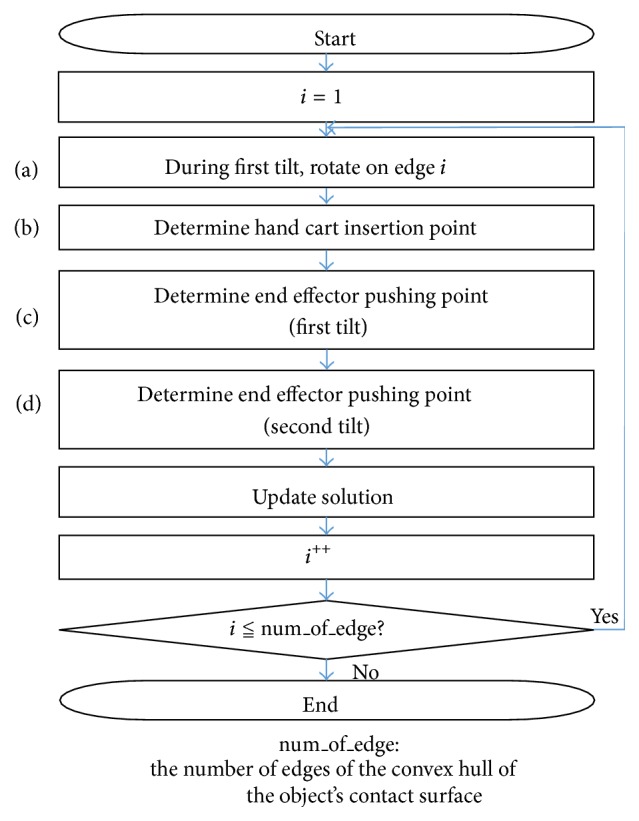
Flowchart of hand cart insertion algorithm.

Figure 4 shows a flowchart of the planned algorithm.

First, in step (a), the center of rotation is determined during the first tilt. During this maneuver, the candidate rotation centers are the edges of the convex hull of the object's contact surface. Each edge of the convex hull is set as the center of rotation (Section 3.3.1), and the algorithm progresses to step (b).

Step (b) of the algorithm determines the hand cart insertion position. Here, for each center-of-rotation candidate selected in step (a), the position for the hand cart insertion is optimized to minimize the objective function (see the next section). Among these candidates, the hand cart insertion position and center of rotation for the first tilt are those yielding the smallest objective function (Section 3.3.2).

Finally, in steps (c) and (d), the end effector pushing points are determined. For the first tilt, the pushing point is determined by the edge of the contact face of the object, determined in step (b) to be the center of rotation. The pushing point (center of rotation) for the second tilt is determined by the straight line connecting the two hand carts inserted in the first tilt (Section 3.3.3).

#### 3.3.1. Selection of Candidates for Rotation Axis of First Tilt

During the first tilt, any edge of the convex hull of the object's contact surface can be the axis of rotation. In the following calculation, the axis of rotation is assumed to be the *i*th edge of the convex hull, and the hand cart insertion position and end effector pushing point are determined. This calculation is performed for each edge of the convex hull (num_of_edge in Figure 4), and the solution yielding the best value for the objective function is selected (corresponding to “Update solution” in Figure 4).

#### 3.3.2. Decision for Insertion Positions of Hand Carts

The hand cart insertion points determine the placements of hand carts that maximally stabilize the final state of the object.

Let the origin of the coordinate system be a point that orthographically projects the object's center of gravity onto the object's contact surface. Now, we align the *z* = 0 plane with the floor surface and define the insertion point of hand cart *i*  (*i* = 1,…, *n*) as **r**_*i*_ = (*r*_*i*_cos⁡*θ*_*i*_, *r*_*i*_sin*θ*_*i*_, 0) on the edge of the contact surface. The center of gravity is set to **r**_*g*_ = (0,0, *r*_*g*_). Here, if *θ*_*i*_ is set, the hand cart insertion point　**r**_*i*_ is uniquely known. As stated above, during the first tilt, two hand carts are inserted parallel to the axis of rotation. Therefore, the number of independent design variables is *n* − 1. We are going to express the problem as an optimization problem composed of constraints and an objective function and then solve it.

*Constraints*

*The Object Does Not Fall Down in the Final State.* The first constraint stipulates that the object does not topple in a particular hand cart arrangement. In other words, the orthogonal projection of the object's center of gravity (the origin) should lie within a polygon formed by *n* hand cart insertion points, as shown in (6):

(6) θi+1−θi<π,θn−θ1>π,i=1,…,n−1,

*The Object Does Not Fall Down during the First Tilt*. If the object is tilted too far during the first tilt, it may topple. This situation is prevented by the limiting condition of the following:

(7) $$\begin{array}{c} \beta =\frac{\pi }{2}-\phi_{O}-\phi_{T}>0, \end{array}$$

where

(8) $$\begin{array}{c} \phi_{T}=\tan^{-1}\left(\frac{H_{c}}{d_{c}-W_{c}/2}\right). \end{array}$$

**Figure 5:**
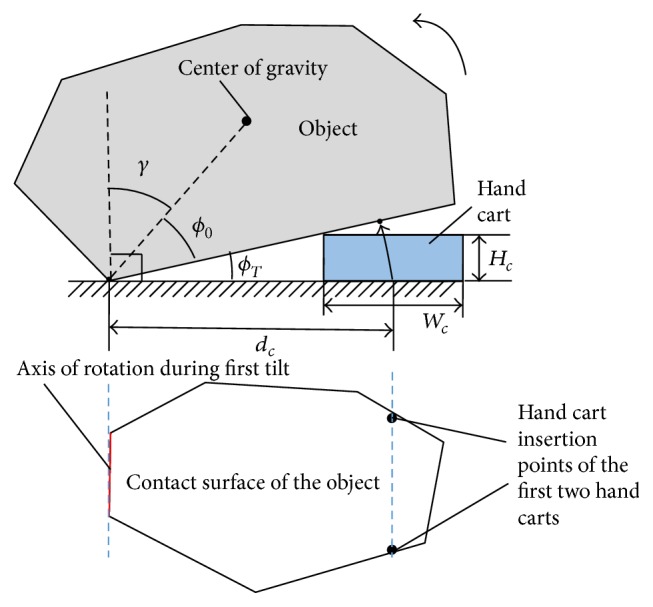
Conditions to avoid toppling of object during tilting.

Here, *H*_*c*_ and *W*_*c*_ are the height and width of the hand cart, respectively. *ϕ*_*O*_ is the angle between the object's center of gravity and the first axis of rotation, as shown in Figure 5. *ϕ*_*T*_ is the angle of inclination required to insert a hand cart during the first tilt. Thus, angle *β* in Figure 5 indicates the allowance of the inclination angle (with respect to the maximum inclination angle) as the object is tilted on some edge. That is, *β* specifies the angle of rotation for inserting the hand cart.

*Collision of Hand Carts*. If the insertion points of the two hand carts are too closely spaced, the carts might collide. This situation is avoided by imposing the condition shown in (4), which specifies a minimum amount of cart separation:

(9) $$\begin{array}{c} \left|r_{i}-r_{j}\right|>w\;\left(i\neq j\right), \end{array}$$

where the constant value *w* specifies the minimum distance needed to prevent the two hand carts from colliding during insertion.

*Objective Function.* The objective function s is computed using (10):

(10) $$\begin{array}{c} s=\min\beta_{i}. \end{array}$$

**Figure 6:**
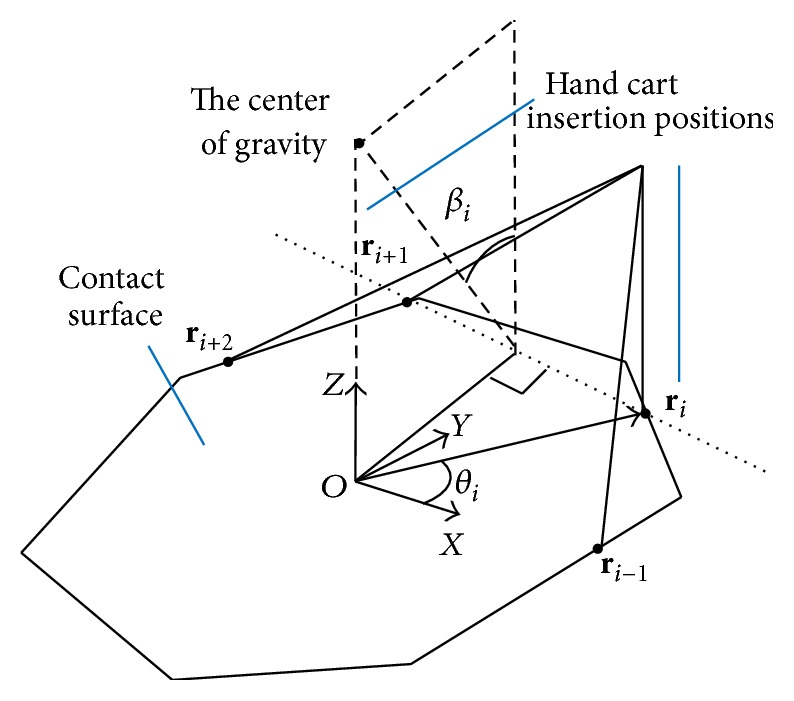
Hand cart insertion points.

Here, we specify the maximum tilt angle of the object around an axis of rotation **r**_*i*_, **r**_*i*+1_, as shown in Figure 6.

Beyond the maximum tilt angle, the object will fall when tilted.

In other words, the objective function *s* is the maximum tilt of an object in the direction of the highest toppling risk in the hand cart arrangement. Thus, the hand cart insertion points are chosen to maximize the objective function equivalently in order to maximize the difficulty of object toppling.

*Solving the Optimization Problem.* Similar to the problem discussed in the previous subsection, the problem can be formulated as a constrained optimization problem. To this end, we define a new objective function (11), whose constraints are expressed as penalty terms:

(11) PI=K1ssum+  K2s+K3C1+C2+C3K3≫K2≫K1

on the condition that

(12) $$\begin{array}{c} s_{\text{sum}}=\overset{n}{\underset{i=1}{\sum }}\beta_{i}, \end{array}$$

(13) $$\begin{array}{c} C_{1}=\left\{\begin{array}{cc} 1 & \left(\text{when}\;\text{constraint}\;\left(6\right)\;\text{is}\;\text{not}\;\text{satisfied}\right)\\ 0 & \left(\text{when}\;\text{constraint}\;\left(6\right)\text{is}\;\text{satisfied}\right), \end{array}\right.\\ C_{2}=\left\{\begin{array}{cc} 1 & \left(\text{when}\;\text{constraint}\;\left(7\right)\;\text{is}\;\text{not}\;\text{satisfied}\right)\\ 0 & \left(\text{when}\;\text{constraint}\;\left(7\right)\text{is}\;\text{satisfied}\right), \end{array}\right.\\ C_{3}=\left\{\begin{array}{cc} 1 & \left(\text{when}\;\text{constraint}\;\left(9\right)\;\text{is}\;\text{not}\;\text{satisfied}\right)\\ 0 & \left(\text{when}\;\text{constraint}\;\left(9\right)\text{is}\;\text{satisfied}\right). \end{array}\right. \end{array}$$

In (12), *s*_sum_ sums all the indices *β*_*i*_ that indicate the difficulty of object toppling. This term guarantees a unique solution to the problem.

The objective function *PI* is minimized by a set of design variables *θ*_1_ ~ *θ*_*n*_. With (11), we can obtain the solution that minimizes *s*_sum_ from among the solutions minimizing *s*, while satisfying constraints (6), (7), and (9). A feasible solution is rapidly obtained using a multistart local search method.

#### 3.3.3. Decision for Pushing Positions of End Effector

*Constraints*

*Payload of the End Effector.* The first constraint restricts the acting force of the end effector to within the transportable weight and is given by

(14) $$\begin{array}{c} \left|f_{y}\right|<F_{\max y},\\ \left|f_{z}\right|<F_{\max z}. \end{array}$$

Here, *F*_max⁡*y*_ and *F*_max⁡*z*_ are the limit of the pushing force of the tilting robot and the transportable weight in the vertical direction of the end effector, respectively. These forces are constants and are determined by the tilting robot specifications.

*Slippage between the Object and Environment or End Effector.* The second constraint stipulates that no slippage occurs between the object and the floor or end effector and is given by

(15) $$\begin{array}{c} S_{\text{fric}}\left(F\right)=\min\left(\delta_{1},\delta_{2},\delta_{3}\right)>0, \end{array}$$

given that

(16) $$\begin{array}{c} \delta_{i}=\tan^{-1}\mu_{e}-\cos^{-1}\left(\frac{R_{i}\cdot e_{z}}{\left|R_{i}\right|}\right)\;\left(i=1,2\right),\\ \delta_{3}=\tan^{-1}\mu_{f}-\cos^{-1}\left(\frac{F\cdot n_{r}}{\left|F\right|}\right). \end{array}$$

Here, **e**_**z**_ is a unit vector in the *z* direction, *μ*_*e*_ is the friction coefficient between the floor and object, and *μ*_*f*_ is the friction coefficient between the end effector and object. The normal vector **n**_**r**_ faces inward toward the plane of the force point **P**_**r**_. If the pushing point lies on the ridgeline of the object, **n**_**r**_ is normal to the tip face of the end effector. In other words, *S*_fric_ specifies the slipperiness between the object and the floor during tilting (*δ*_1_, *δ*_2_) or the slipperiness between the object and the end effector (*δ*_3_), whichever is smaller. This constraint needs to be satisfied in both the first and second tilts.

*Objective Function.* The objective function is given by

(17) $$\begin{array}{c} S_{\text{fric}}\left(F\right)⟶\max. \end{array}$$

Equation (17) introduces the end effector pushing point to maximize the difficulty of slippage.

*Solving the Optimization Problem*. The design variable for this problem is the end effector pushing point **P**_*r*_, as explained in Section 3.2. To simplify the problem, we generate discretized candidate points on the object surface and calculate a solution.The procedure is outlined below.

 (*1) Generate Candidate Points*. To calculate the pushing point, candidate points for **P**_**r**_ are generated on the object surface across a uniform grid. Because the movable area of the end effector is limited, the candidate points must fulfill the constraint shown in

(18) $$\begin{array}{c} Z_{\min}<p_{rz}<Z_{\max}. \end{array}$$

Here, *Z*_min⁡_ and *Z*_max⁡_ are the upper and lower boundaries, respectively, of the movable area of the end effector in the vertical direction.

 (*2) Determine the End Effector Pushing Point*. *S*_fric_ is solved at each candidate point. Among the candidate points satisfying (14) and (15), the end effector pushing point is the end effector point with the highest value.

The end effector pushing point in the second tilt is similarly determined, with a couple of minor differences. Here, the object contacts the hand cart along the axis of rotation, and the object tilts only in the region where the hand cart is inserted. In other words, the same method can be adopted for both the first and second tilts, simply by changing the friction coefficient for the axis of rotation and the direction of the applied weight.

### 3.4. Simulation

**Figure 7:**
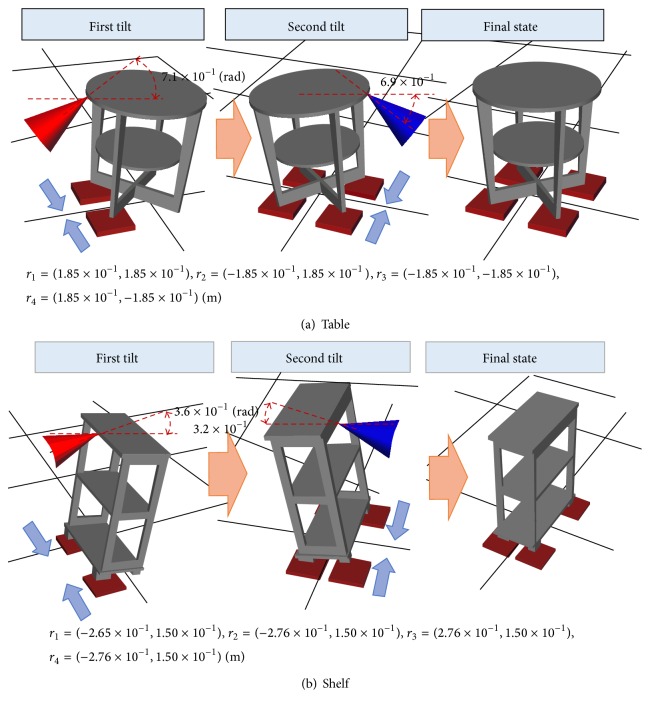
Simulation results. The coordinate values of the four insertion positions for hand carts are described below each figure (the origin of the coordinate system is located at the object's center of gravity). The red and blue cones show the end effector pushing forces for the first and second tilts, respectively.

In this section, we show how a simulation was used to investigate the capability of the proposed algorithm to insert hand carts. The friction coefficients were as follows: (between the object and floor) 0.4, (between the object and end effector) 0.5, (between the object and hand cart) 0.5, and (between the hand cart and floor) 0.3. The table and shelf shown in Figure 7 were transported by four hand carts. The transported objects, which were generated using a triangular mesh, were geometric models of the furniture items used in the real-time experiment described in Section 4. The hand cart insertion positions were optimized using parameters *K*_3_ = 1000000, *K*_2_ = 100, and *K*_1_ = 1.

The simulation results are shown in Figure 7. For the shelf, the center of the long side of the top was first pushed in the upward diagonal direction. The second tilt lifted the opposite edge in the upward diagonal direction.

Calculations were performed on a computer with an Intel Core i5-580M 2.67 GHz CPU. The table and shelf simulations consumed 4.68 × 10 s and 1.71 × 10 s of runtime, respectively. The number of optimization calculations for the hand cart insertion points depended on the number of axis-of-rotation candidates. The number of calculations was also proportional to the number of edges on the convex hull of the object. Therefore, the calculation time was governed by the shape of the object.

Appropriate solutions were generated even for geometric models based on real objects, which demonstrated the validity of the algorithm.

## 4. Experiment

This section shows how the transport method was tested on a real-time robot system in a real environment. An end effector was designed to tilt the object, and each robot was designed to accurately adjust its position and orientation based on self-localization. We investigated whether the system could maneuver real objects and the degree of response to potential errors.

### 4.1. Hardware

**Figure 8:**
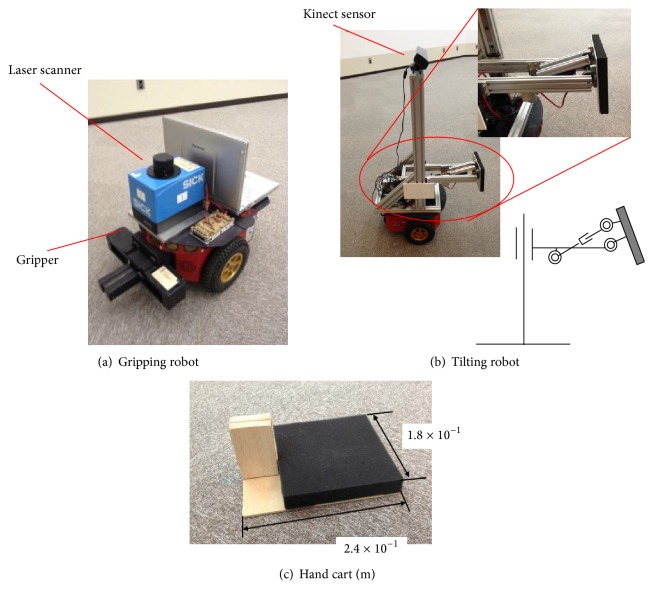
Hardware.

The hardware of the robot system is shown in Figure 8. The gripping robot was a Pioneer P3-DX (length 3.81 × 10^−1 ^m, width 4.55 × 10^−1 ^m; see Figure 8(a)). This robot was equipped with parallel grippers in the forward direction, by which it grasped and moved a hand cart.

The tilting robot (Figure 8(b)) pushed and tilted the object using a two-degree-of-freedom end effector. The design of this end effector is discussed below.

The robot pushed on a part of the object to tilt it. Because less force needs to be applied to an object at a higher pushing position, structures that can withstand larger reactive forces at high points are desirable for tilting heavy objects. Moreover, the payload generally decreases with an increase in the degrees of freedom of the end effector, which increases the complexity of the mechanism. For this reason, the degrees of freedom of the end effector should be maintained as small as possible. Thus, we specified the following requirements for the end effector.

- The mechanism can withstand a large reactive force at a high point.
- It should have a low degree of freedom.

**Figure 9:**
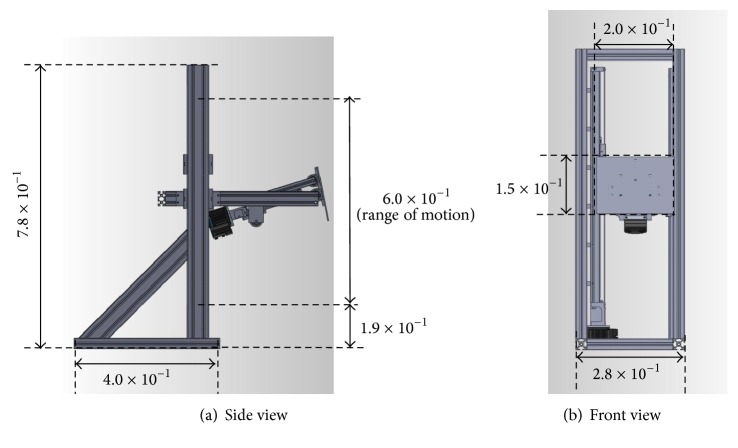
Dimensions of end effector (m).

Here, we allocated two degrees of freedom for the end effector: the pitch rotation of the end effector tip and vertical translation. The constructed end effector incorporated two linear motion mechanisms driven by ball screws (Figure 8(b)). The reactive force was reduced by ball screw friction and transferred to the motor, rendering the mechanism resistant to a large reactive force. In addition, because the prismatic joint of the tipping rotation was also driven by a ball screw, the mechanism was resistant to the moments applied in the rotational direction of the tip. The dimensions of the constructed end effector, loaded onto the upper portion of the mobile robot, are shown in Figure 9. With the end effector attached, the length and width of the robot were 7.5 × 10^−1 ^m and 4.6 × 10^−1 ^m, respectively, fulfilling the size specifications discussed in Section 1.

Handles were provided for the hand carts, and their upper surfaces were equipped with 1.8 × 10^−1 ^m flexible square parts (Figure 8(c)) to increase the adhesion with the target object, balance the load from the object to each hand cart, and transport the object safely by allowing the hand carts to move synchronously. The wheels of the hand cart were replaced with smooth boards to keep the height of the cart (*H*_*c*_) as small as possible, which was important for the stable tilting of the objects.

### 4.2. How Each Robot Modifies Its Own Position

In the proposed method, the mobile robots perform three tasks: tilting the object, grasping the hand cart, and inserting the hand cart. Before realizing these tasks, the mobile robots must determine their own relative positions and orientations, along with those of the hand carts and object. To this end, they perform the following localization.

**Figure 10:**
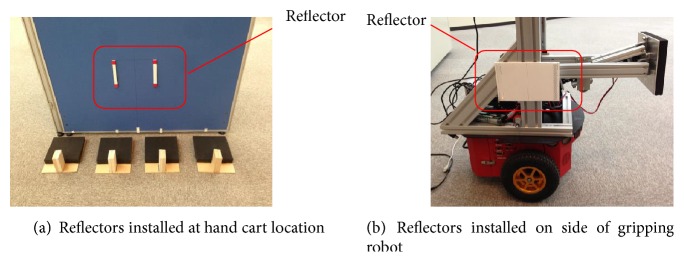
Reflectors.

The gripping robot corrects its position using a laser scanner and reflectors. When grasping the hand cart, it laser-scans the positions of the reflectors installed at the locations of the hand carts (Figure 10(a)) and corrects its position based on this information. Here, the relative positions/orientations of the reflectors and the initial positions/orientations of the hand carts are fixed, and the information is given to the robots in advance, which means the robots can grasp the hand carts by measuring the positions and orientations of the reflectors. When inserting a hand cart, it detects the reflectors installed on the side of the gripping robot (Figure 10(b)).

**Figure 11:**
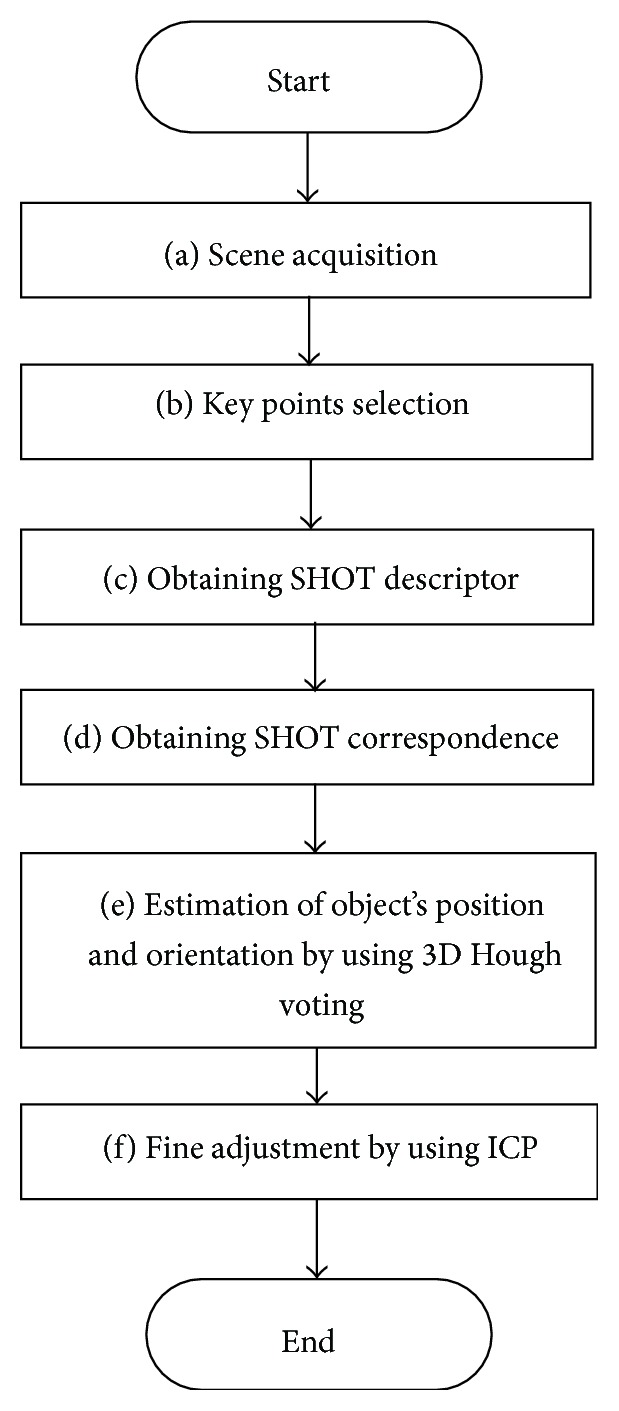
Flowchart of object recognition.

**Figure 12:**
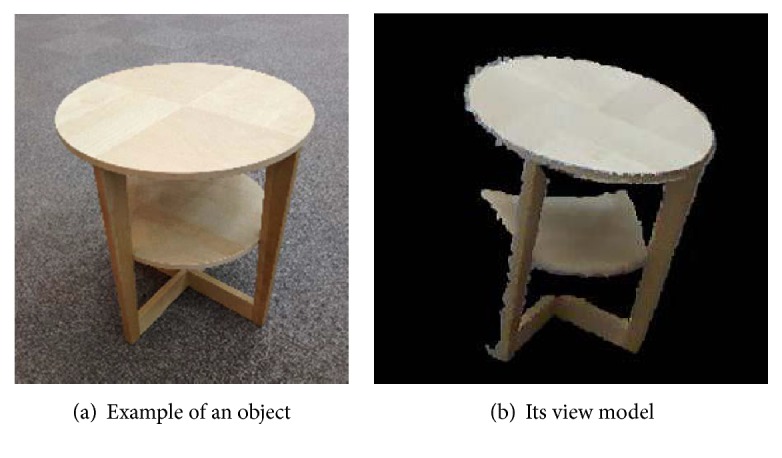
Example of transported object and its view model.

The gripping robot performs object recognition and localization, as shown in Figure 11, using a Kinect sensor, which collects point group information about the object (Figure 11(a)). The gripping robot uses an object view model (Figure 12), which is prepared in advance, to estimate the position and orientation of the object. In estimating the position and orientation of the object, the features of the object are picked up as key points (Figure 11(b)). They are transformed into a SHOT descriptor [21], which consists of the positions of the key points and the normal vectors near the points (Figure 11(c)) and obtains the correspondence between the key points of the obtained sensor data and those of the model data (Figure 11(d)). The position and orientation of the object are roughly estimated using 3D Hough voting [22] (Figure 11(e)). These estimated values are processed using an ICP algorithm [23] to more correctly determine the position and orientation of the object (Figure 11(f)). Because the methods given in [21, 22] are robust to occlusions, this method is appropriate in real environments where only a portion of a large object is visible for position estimation.

**Figure 13:**
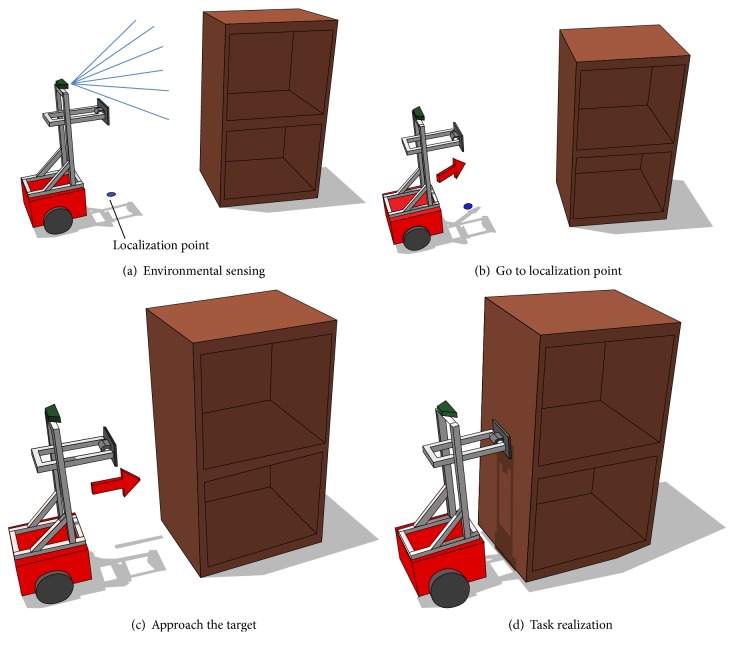
Localization process.

It also estimates its own position from point group information gathered immediately before tilting the object, as shown in Figure 13. First, the robot moves to a localization point using its own odometry sensor, which has estimation errors (Figure 13(a)). Second, by measuring the relative position and orientation of the object with the above-mentioned procedure, the robot can detect positioning error and move to the localization point (Figure 13(b)). Third, after reaching the localization point, the robot approaches the target (Figure 13(c)). Finally, the robot realizes the task (Figure 13(d)).

For each task, the positions for localization are decided in advance (a set distance behind the operation position). The robots then advance forward and perform each action.

### 4.3. Environmental Setting

**Figure 14:**
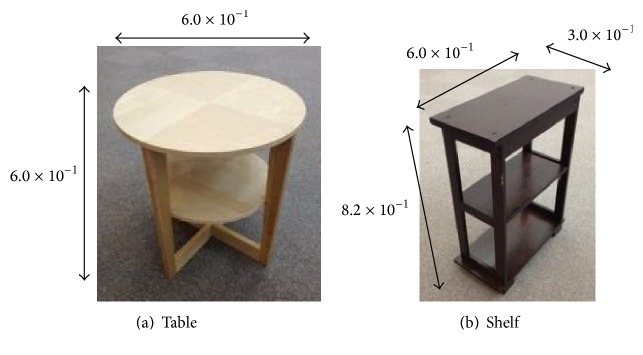
Objects for experiments (m).

The payload objects were the table (7.8 kg) and shelf (10.6 kg) shown in Figure 14. The hand cart insertion positions and end effector pushing positions were as described in Section 3.

### 4.4. Experimental Results

**Figure 15:**
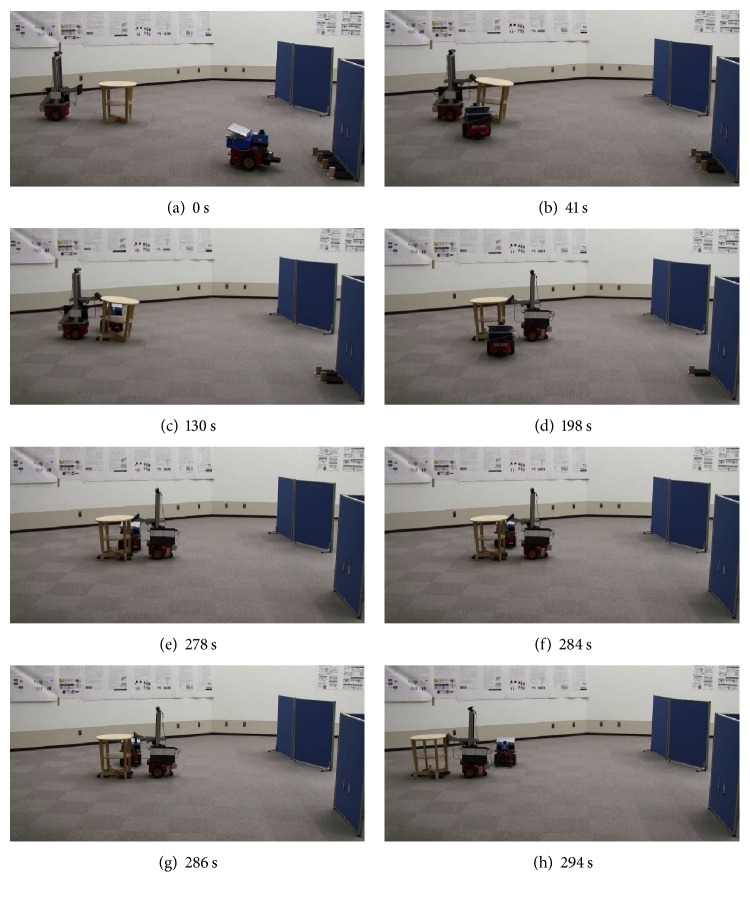
Transporting table.

**Figure 16:**
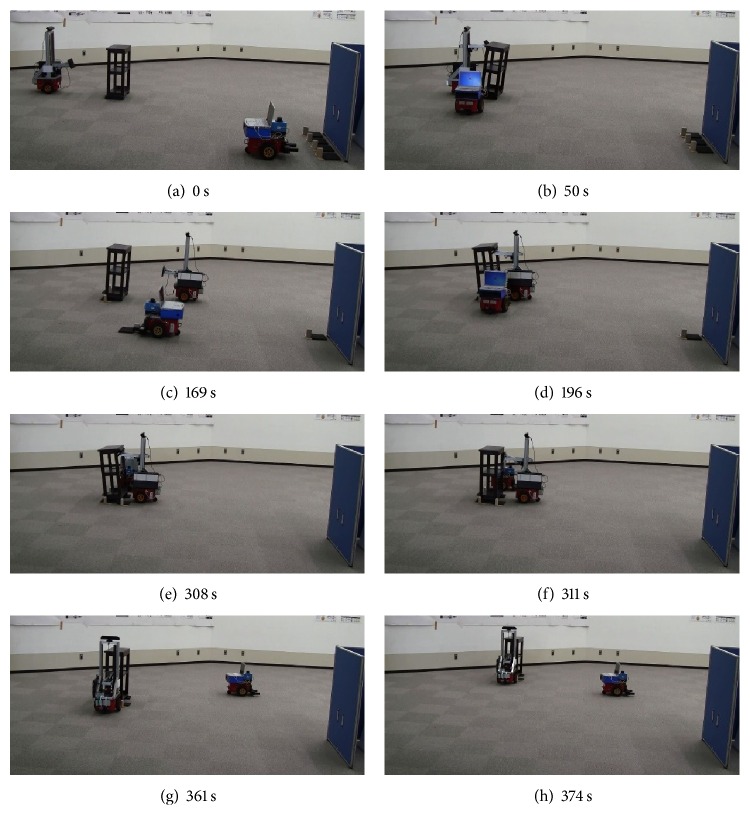
Transporting shelf.

The experiment was performed three times for each of the two objects. The results are shown in Figures 15 and 16. The robots succeeded in tilting the object, grasping the hand carts, inserting the hand carts, loading the objects onto the hand carts, and transporting them to their destinations, by correcting their positions, regardless of motion errors. There was no control problem with the handcarts while transporting the objects to their destinations. Please note that the destination of the table and that of the shelf are different (from right to left in the case of the table and from the near side to far side in the case of the shelf).

The tasks consumed an average of 294 and 374 s for the table and shelf, respectively. Summing the time required for the first and second tilts, the time consumed in estimating the position and orientation of the table was 15 s (5% of the overall work time), whereas that for the shelf was 31 s (8% of the overall work time).

Considering the grippers on the p3-dx mobile robot used to transport these objects, we could specify the required number of robot units in the cooperative grasping method. Given that the payload of the grippers was 2.5 kg, at least four and five mobile robots would be required to move the table and shelf, respectively. Thus, for the objects used in this experiment, the proposed method reduced the number of required mobile robots with 50–60%.

We next consider the robot localization errors. We calculated the average errors for each robot during object tilting and hand cart insertion.

**Figure 17:**
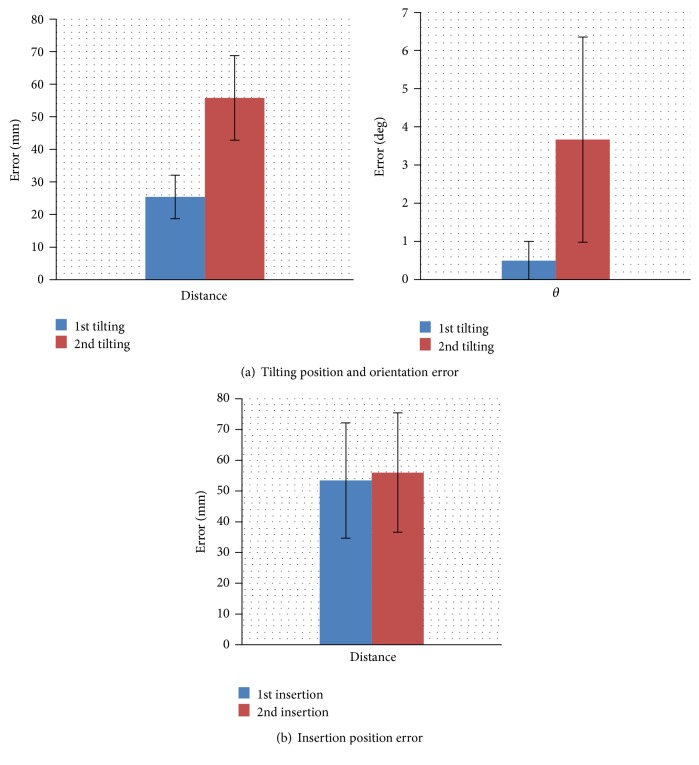
Localization error of two mobile robots.

Figure 17(a) displays the localization errors of the tilting robot immediately before tilting the object. The left side of the figure shows the distance error of the tilting robot—defined as the distance between the end effector pushing position (supplied from the input) and the true center of the end effector tip. The right side shows the average error in the angle of the tilting robot: the horizontal angle generated by the direction of the end effector active force (supplied from the input) and the true direction of the end effector tip. Prior to tilting the object, the tilting robot generated average position and orientation errors of 2.0 × 10^−2^ ~ 6.0 × 10^−2^ m and 0.9 × 10^−2^ ~ 6.1 × 10^−2^ rad, respectively. Because the end effector tips were rectangular, with edges measuring 2 × 10^−1 ^m and 1.5 × 10^−1 ^m, the object could be reliably tilted when the errors were in the above range. These results showed that it was effective to apply the position control to the end effector of tilting robot.

Figure 17(b) displays the errors in the inserted hand cart position. Errors with a magnitude of 10^−2 ^m (more specifically, average error = 5.5 × 10^−2 ^m) did not prevent the object from being loaded onto the hand cart.

These results demonstrated that the positional correction methods for each robot functioned appropriately, and each action was performed with sufficient positional precision for loading an object onto a hand cart. Thus, the proposed method is potentially applicable to a real environment.

## 5. Conclusion

This study proposed a method by which mobile robots can load objects onto hand carts for transport. In the existing methods for object transport, it is difficult to lift large objects because of the relative powerlessness of mobile robots. In contrast, the hand carts adopted in the proposed method reduce the friction between the object and the floor and also the force necessary to transport the object. Because objects are loaded onto hand carts by tilting rather than lifting, this method is expected to allow a minimum number of robots to transport larger and heavier objects.

We reduced the problem of planning mobile robot actions during loading to an optimization problem of operation procedures and demonstrated the validity of the proposed algorithm in a simulation.

We then tested the proposed method in a real-world experiment using customized hardware. We also constructed and assessed a method for robots to perform position and orientation correction. In this experimental environment, the number of robots required for transporting an object could be reduced with 50–60%. In addition, the proposed planning algorithm and robot system enabled objects to be transported on hand carts in real environments.

In future work, we will consider improvements to the hand carts. Because the hand carts were replaced with boards in this experiment, the friction reduction was small. In addition, the hand cart supporting the object might slip during the hand cart insertion operation. The applicability of the proposed method might be expanded by using wheeled hand carts equipped with brakes. This paper has focused on loading an object onto a hand cart. Another future task is a localization problem while unloading objects from hand carts.
